# Veterinary Students Have a Higher Risk of Contracting Cryptosporidiosis when Calves with High Fecal *Cryptosporidium* Loads Are Used for Fetotomy Exercises

**DOI:** 10.1128/AEM.01250-20

**Published:** 2020-09-17

**Authors:** Daniel Thomas-Lopez, Luise Müller, Lasse S. Vestergaard, Mette Christoffersen, Anne-Marie Andersen, Pikka Jokelainen, Jørgen Steen Agerholm, Christen Rune Stensvold

**Affiliations:** a European Public Health Microbiology Training Programme (EUPHEM), European Centre for Disease Prevention and Control (ECDC), Stockholm, Sweden; b Laboratory of Parasitology, Department of Bacteria, Parasites & Fungi, Infectious Disease Preparedness, Statens Serum Institut, Copenhagen, Denmark; c Department of Infectious Disease Epidemiology and Prevention, Infectious Disease Preparedness, Statens Serum Institut, Copenhagen, Denmark; d Department of Veterinary Clinical Sciences, University of Copenhagen, Taastrup, Denmark; Rutgers, The State University of New Jersey

**Keywords:** *Cryptosporidium*, outbreak, zoonosis, One Health, diarrhea, cattle

## Abstract

An outbreak of cryptosporidiosis among veterinary students performing fetotomy exercises on euthanized calves took place in September 2018 in Denmark. A prospective cohort investigation was performed to identify risk factors and provide guidance for preventing outbreaks of cryptosporidiosis in this setting. Ninety-seven students attended the fetotomy exercises and completed a questionnaire about symptoms and potential risk behavior. Real-time PCR was used to detect *Cryptosporidium* spp. in stool samples from students and to quantify the fecal parasite load in the calves used for the exercises. *gp60* subtyping was carried out for the *Cryptosporidium*-positive samples. Our case definition was based on participation in a fetotomy exercise, reported symptoms, and laboratory results. Eleven laboratory-confirmed or probable cases (11%) were identified in two outbreaks during the prospective study period, with attack rates of 4/10 (40%) and 7/9 (78%), respectively. The risk factors for cryptosporidiosis we identified were performing the exercise on a diarrheic calf, reporting visible fecal contamination on the personal protective equipment (PPE), and reporting problems with PPE during the exercise. Cryptosporidium parvum IIaA15G2R1 was detected in both cases and calves. A significantly higher proportion of the calves aged 7 days old and above were positive compared with younger calves. Furthermore, a high fecal *Cryptosporidium* load in a calf was associated with a higher probability of an outbreak among the students. Based on our results, using noninfected calves for the exercises, appropriate use of PPE, and thorough hand hygiene are recommended to reduce the risk of contracting cryptosporidiosis in connection with fetotomy exercises.

**IMPORTANCE** *Cryptosporidium* spp. can cause severe diarrhea in infected individuals. Cryptosporidium parvum is zoonotic, and cattle are the main reservoir. In several countries, outbreaks of cryptosporidiosis have occurred in veterinary students after handling calves. We carried out a 1-year-long prospective study to investigate the occurrence of these recurrent cryptosporidiosis outbreaks in Denmark. Our investigation used a One Health approach and combined comprehensive epidemiological approaches and laboratory methods applied to both students and calves in the setting of the fetotomy exercises. Two outbreaks took place during the study period; additionally, we retrospectively identified two more suspected outbreaks prior to the study period. The results illustrated a high risk of contracting cryptosporidiosis among veterinary students in the setting of the fetotomy exercises, especially when using calves with high fecal *Cryptosporidium* loads. Our data can be used to inform future efforts to prevent transmission of Cryptosporidium parvum to students during fetotomy exercises.

## Abstract (precis)

*Cryptosporidium* spp. can cause severe diarrhea in infected individuals. Cryptosporidium parvum is zoonotic, and cattle are the main reservoir. In several countries, outbreaks of cryptosporidiosis have occurred in veterinary students after handling calves. We carried out a 1-year-long prospective study to investigate the occurrence of these recurrent cryptosporidiosis outbreaks in Denmark. Our investigation used a One Health approach and combined comprehensive epidemiological approaches and laboratory methods applied to both students and calves in the setting of the fetotomy exercises. Two outbreaks took place during the study period; additionally, we retrospectively identified two more suspected outbreaks prior to the study period. The results illustrated a high risk of contracting cryptosporidiosis among veterinary students in the setting of the fetotomy exercises, especially when using calves with high fecal *Cryptosporidium* loads. Our data can be used to inform future efforts to prevent transmission of Cryptosporidium parvum to students during fetotomy exercises.

## INTRODUCTION

Parasites of the genus *Cryptosporidium* cause food- and waterborne outbreaks and are among the leading causes of diarrhea in children worldwide (1–3). In healthy adults, different *Cryptosporidium* spp. may cause severe diarrhea and other gastrointestinal symptoms that can last for up to 2 weeks (4, 5), while in immunocompromised individuals, cryptosporidiosis can develop into chronic or extraintestinal infections (5, 6). Together with Cryptosporidium hominis, Cryptosporidium parvum is commonly involved in human infections (5, 6). Cattle are the main reservoir of C. parvum (7), and the infection is common in calves worldwide (6–10). Infected calves may develop diarrhea and shed up to 10^7^ oocysts per gram of feces (11).

Outbreaks of cryptosporidiosis in veterinary students have been widely reported and associated with direct handling of calves during academic training (12–17). Some of these outbreaks have been linked to fetotomy exercises (14). A fetotomy is an obstetrical procedure involving intrauterine dissection of a dead fetus to enable vaginal extraction. The procedure is mainly applied to cows and mares. In the veterinary curriculum, this procedure can be practiced on a dead calf placed inside an artificial uterus (see Fig. S1 in the supplemental material).

In September 2018, the Statens Serum Institut (SSI) in Copenhagen, Denmark, was contacted by the Department of Veterinary Clinical Sciences at the University of Copenhagen, Denmark, after several students had reported symptoms compatible with cryptosporidiosis upon attending a fetotomy exercise. As similar outbreaks had happened previously (18), a multidisciplinary team including veterinarians, medical doctors, parasitologists, epidemiologists, infection control specialists, and public health experts was established following a One Health approach (6, 20, 21) in order to carry out an investigation to identify key risk factors for the outbreaks and to suggest relevant preventive measures.

## RESULTS

### Sequence of events, outbreak investigation, and interventions.

**FIG 1:**
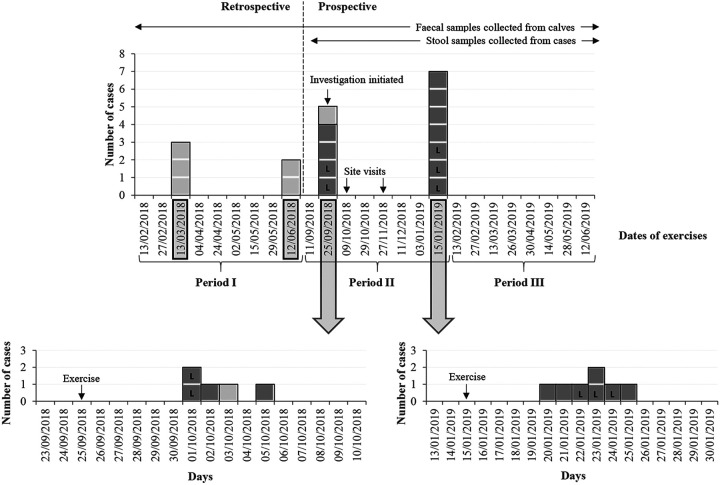
Number of cases of cryptosporidiosis among veterinary students linked to the fetotomy exercises at the University of Copenhagen, Denmark, from January 2018 to June 2019. Laboratory-confirmed cases (L), probable cases (dark gray), and suspected cases (light gray). Dates of outbreaks among the veterinary students are highlighted in boxes. The starting point of the investigation and the exercises observed (site visits) are indicated. The three periods of the investigation and whether information was obtained retrospectively or prospectively are specified. Time frames during which samples were collected from calves or from cases are indicated. Detailed timelines of the two outbreaks investigated, with the dates of onset of reported symptoms, appear underneath the main timeline.

Twenty-five fetotomy exercises took place from January 2018 to June 2019 (periods I to III) (Fig. 1; Table S1). The prospective cohort investigation was initiated in September 2018 and covered periods II and III.

For period I (January to August 2018), data retrieved retrospectively suggested that outbreaks had taken place in March 2018 and June 2018, with three and two suspected cases (participated in the exercise and informed the university staff of symptoms compatible with cryptosporidiosis), respectively. Nine fetotomy exercises had taken place, and 45 calves had been used in this period (Table S1). Further information about the students, symptoms, and behavior during the exercise was not available for period I.

Period II began with the outbreak in September 2018, which marked the initiation of the prospective cohort study. Five cases among 10 students (attack rate, 50%) were identified (Fig. 1)—two laboratory-confirmed (participated in the exercise, reported compatible symptoms in the questionnaire, and tested positive for *Cryptosporidium* spp.), two probable (participated in the exercise and reported compatible symptoms in the questionnaire), and one suspected case. Two site visits to observe the fetotomy exercises took place in October and November 2018, respectively. It was observed that for personal protective equipment (PPE), the students used disposable long-sleeved full-length plastic gowns, short and long disposable plastic gloves, surgical caps, and surgical masks. The students used their own rubber boots as footwear. Duct tape was used around the wrists and around the waist. The clean disposable PPE was stored in original boxes, uncovered, on a table in the exercise room. After use, the fetotomy instruments were placed in a sink that was located in the exercise room and that was also used for hand washing. At the end of the exercise, the students left the room after doffing the PPE, washing their boots, and carrying out hand hygiene with soap and water as well as with alcohol gel. The students changed back to their own clothing and footwear in a separate building where they had lockers and shower facilities.

Based on the observations and data collated, several infection control interventions were proposed, including advice on the type, use, and storage of PPE. Various actions were taken; for instance, a designated area for PPE storage and donning was allocated outside the fetotomy exercise room, and the students were provided with more detailed instructions for the use of PPE. The sink used for hand washing was no longer used for cleaning the instruments.

Despite these interventions, another outbreak took place in January 2019 (Fig. 1). Seven cases—three laboratory-confirmed and four probable cases—among nine students (attack rate, 78%) were identified. This outbreak marked the end of period II, which comprised eight fetotomy exercises involving 87 students and 37 calves (Table S1).

In February 2019, the hands-on exercise was made voluntary for the students to attend. This marked the beginning of period III (Fig. 1), during which 31 (46%) of the 68 students who had initially registered for the course participated in the exercise (Table S1). In each exercise, there were on average 4 students, and the number of calves used per exercise was proportionally reduced to 1 to 3. Eleven calves were used in eight fetotomy exercises. No cases of cryptosporidiosis were identified among the students during this period.

### Epidemiological findings and questionnaire results.

Overall, 119 students attended the fetotomy exercises during periods II and III (Table S1), 97 of whom completed the questionnaire (response rate, 81.5%). The students were between 22 and 40 years old (mean age, 25.9 years) and the majority were female (82.5%) (Table 1).

**TABLE 1:** Descriptive characteristics of the veterinary students (*n* = 97) and cases of cryptosporidiosis^*a*^ (*n* = 11)

Characteristic	Data for male students	Data for female students	Total
Number of participants (%)	17 (17.5)	80 (82.5)	97 (100)
Age of participants in yrs, range (mean; median)	22–40 (27; 25)	22–37 (25.7; 25)	22–40 (25.9; 25)
Number of cases^*a*^ (%)	3 (27.3)	8 (72.7)	11 (100)
Age of cases, range (mean; median)	25 (25; 25)	23–31 (26; 25.5)	23–31 (25.7; 25)
Information about symptoms as reported by the 11 cases^*a*^			
Incubation time, days (mean; median)			5–10 (7.5; 7)
Duration of symptoms, days (mean; median)			1–14 (5.4; 5)
Abdominal cramps, no. of cases (%)			10 (90.9)
Diarrhea, no. of cases (%)			9 (81.8)
Nausea, no. of cases (%)			7 (63.6)
Fever, no. of cases (%)			7 (63.6)
Vomiting, no. of cases (%)			1 (9.1)
Other symptoms^*b*^, no. of cases (%)			3 (27.3)

a Laboratory-confirmed and probable cases of students who participated in a fetotomy exercise at the University of Copenhagen, Denmark, from September 2018 to June 2019, and completed the questionnaire.

b Headache was specified by all three cases.

The 11 veterinary students (4 in September 2018 and 7 in January 2019) who met the definition of “laboratory-confirmed” or “probable” case did not differ demographically from the other students (Table 1). Three of the cases were reportedly absent from teaching for between 2 and 7 days due to the illness. One case reported seeking medical care; none were hospitalized. In all, 3 cases (3/11, 27%) and 29 other respondents (29/86, 31%) reported contact with cattle during clinical night shifts in the days following the exercise, while the rest reported no contact with cattle besides the fetotomy exercise. Most of the students reported having received information on hygienic measures and *Cryptosporidium* infection from the supervisor prior to the fetotomy exercise. This included an overview of the pathogen, the risk of infection in connection with the exercises, and how the students should behave in relation to the exercises. Altogether, 72 (74%) of the students reported nervousness about the possibility of getting sick after the exercise.

Allocating designated areas (for clean PPE storage, donning, and handwashing) did not reduce the risk of cryptosporidiosis among the students in comparison with the time period before this intervention (Table S2). Based on the epidemiological study, three risk factors associated with contracting cryptosporidiosis were identified (Table 2), performing the fetotomy on a calf with diarrhea, having the PPE visibly contaminated with feces, and experiencing problems with the PPE such as breaking or loosening of the plastic gown. Using soap for hand washing, either alone or with alcohol gel after doffing the disposable gown and long gloves, was a protective factor (Table 2). The complete univariable analysis can be found in Table S2.

**TABLE 2:** Statistically significant determinants for cryptosporidiosis in the veterinary students^*a*^*^,^*^*b*^

Factor	Factor present?	No. of cases	Total^*c*^	AR (%)	RR	95% CI	*P* value
Risk factors							
Performing the fetotomy on a calf with diarrhea	Yes	9	48	18.75	All cases	NA	0.023
	No	0	25	0			
Having PPE visibly contaminated with feces	Yes	8	29	27.59	11.31	1.49–85.58	0.003
	No	1	41	2.44			
Experiencing problems with PPE	Yes	9	48	18.75	4.59	1.05–20.17	0.028
	No	2	49	4.08			
Protective factors							
Using soap and alcohol gel for hand hygiene	Yes	3	49	6.12	0.21	0.06–0.74	0.012
	No	7	24	29.17			
Using only soap for hand hygiene	Yes	0	15	0	0	NA	0.031
	No	7	24	29.17			

a Data are for veterinary students who attended a fetotomy exercise at the University of Copenhagen, Denmark, from September 2018 to June 2019, and completed the questionnaire (*n* = 97).

b AR, attack rate; RR, risk ratio; CI, confidence interval; NA, not applicable; PPE, personal protective equipment.

c Those who replied “I don’t know/I don’t remember” were omitted from the analysis for the specific determinant.

### Microbiological findings.

The five laboratory-confirmed cases tested positive for *Cryptosporidium* (median threshold cycle [*C_T_*], 31.8; interquartile range **[**IQR], 26.5 to 34.3), which were subtyped as C. parvum IIaA15G2R1. None of the cases were positive for any of the other pathogens that they were tested for (data not shown).

**FIG 2:**
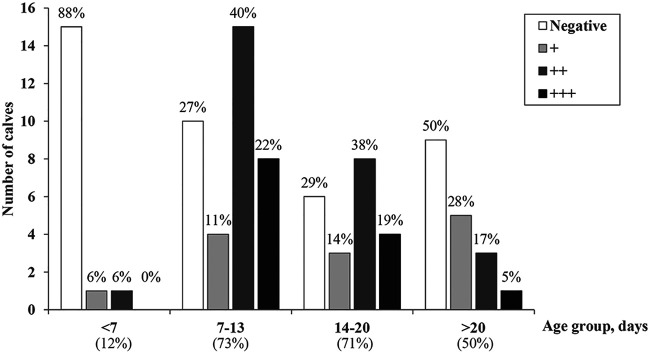
Number of calves used for fetotomy exercises at the University of Copenhagen, Denmark, from January 2018 to June 2019 by age group and fecal *Cryptosporidium* load (quartiles based on real-time PCR *C_T_* values): “negative,” “weakly positive” (+), “positive” (++), and “highly positive” (+++). The percentage of positive calves within each age group is indicated.

Altogether, 53 out of the 93 (57%) calves used in the exercises during periods I to III tested positive for *Cryptosporidium* (median *C_T_*, 28.9; IQR, 23.8 to 35.0) (Table S1). The proportion of positive calves among those that were ≥7 days old was significantly higher than that among younger calves (*P < *0.001). Moreover, the fecal *Cryptosporidium* load of the ≥7-day-old calves was higher than that of the younger calves (Fig. 2). Subtyping was successful for 34 of the 53 positive samples from calves, and in all of them, C. parvum IIaA15G2R1 was detected.

**FIG 3:**
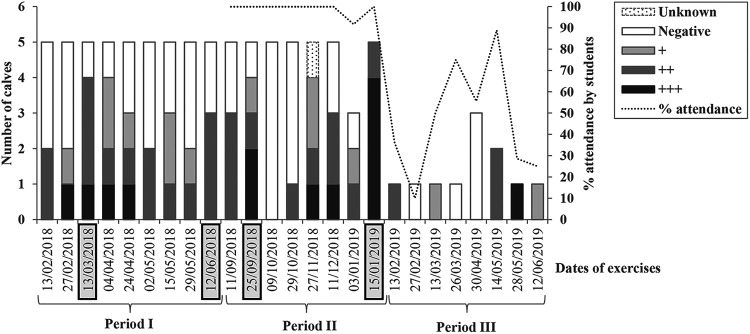
Number of calves used for fetotomy exercises at the University of Copenhagen, Denmark, from January 2018 to June 2019 by date of the exercise and fecal *Cryptosporidium* load (quartiles based on real-time PCR *C_T_* values): “negative,” “weakly positive” (+), “positive” (++), and “highly positive” (+++). Dates of outbreaks among the veterinary students are highlighted in boxes. The percentage of students attending each of the hands-on exercises is indicated by a dashed line.

The fecal *Cryptosporidium* loads of the calves differed by exercise (Fig. 3). The probability of an outbreak occurring was associated with the level of infection and shedding among the calves in the room, with a dose-response relationship being evident (Table 3). Furthermore, no single calf appeared as the sole cause of any of the outbreaks, as the cases did not cluster by the subgroups of students working with a specific calf (data not shown).

**TABLE 3:** Risk ratio (RR) analysis of the probability of a cryptosporidiosis outbreak among veterinary students, depending on the fecal *Cryptosporidium* load of the calves used for fetotomy exercises^*a*^

Fecal *Cryptosporidium* load category	No. of calves	No. of calves used on an outbreak day	Proportion of calves used on an outbreak day (%)	RR	95% CI	*P* value
+++ (*C_T_* < 23.8)	13	7	53.85	5.38	1.87–15.50	0.002
++ (*C_T_* 23.8–35.0)	27	8	29.63	2.96	0.99–8.87	0.054
+ (*C_T_* > 35.0)	13	1	7.69	0.77	0.09–6.28	1.000
Negative	40	4	10	Ref.	Ref.	Ref.

a Ref., reference value; RR, risk ratio; CI, confidence interval.

## DISCUSSION

Our work adds substantially to the knowledge and understanding of recurrent C. parvum outbreaks among veterinary students. We combined an epidemiological cohort investigation over a time frame of more than 1 year using the same quantitative laboratory method and subtyping for the analysis of samples from both the human cases and the calves. In general, other similar investigations carried out to date have included a smaller number of students, were limited to shorter time frames, and/or did not apply quantitative methods (12, 15, 17).

C. parvum subtype IIaA15G2R1 was the only subtype found, and it was identified in samples from both the cases and the calves. This subtype is common in Denmark and worldwide, especially in young cattle (4, 5, 22). Therefore, while the identification of the same subtype supported there being a link between the cases and the calves in our study, it was insufficient to confirm it. For this reason, the combined results from the microbiological and epidemiological approaches were important to reach the conclusions. Subtyping results, together with the time of symptom onset among the cases and the absence of other reported common exposures, supported the link in two separate outbreaks. Furthermore, the quantification of the parasite load in the samples from the calves allowed us to evince a dose-response relationship: a higher level of shedding among the calves in the room corresponded to a higher probability of an outbreak taking place.

Reducing the risk of cryptosporidiosis is challenging due to the high prevalence in calves (7, 9), the resistance of the oocysts to many disinfectants, long survival of the oocysts in the environment, and the low dose required to infect humans; i.e., fewer than 10 oocysts (19, 23). These are challenges that reinforce avoiding the use of infected and shedding calves as the key recommendation. Compared with the calves aged less than 7 days old, a high proportion of the older calves were positive for *Cryptosporidium* spp. and had higher parasite loads. Therefore, using only calves aged below 1 week would decrease the risk of the students becoming infected in the setting of the exercises.

Studies reporting on similar outbreaks elsewhere found that the interaction of the students with diarrheic calves was a risk factor (12, 17) and highlighted the importance of appropriate hygiene and PPE (14, 15). Similarly, in our study, performing the fetotomy on a calf with diarrhea, handwashing without soap, and having visible fecal contamination on the PPE stood out as significant risk factors for cryptosporidiosis. Hence, despite removal of the gastrointestinal tract of the calves before the exercises, students were still exposed to *Cryptosporidium*. The exposure probably happened either while handling the calves at euthanasia or when performing the fetotomy exercise because of the very close contact with the calves. The calves were kept closely together in a confined space prior to the exercise, so if a calf had diarrhea, the hair coat of other calves might have been contaminated and therefore pose a risk to the students handling them. This may explain why a specific calf could not be identified as the sole source of any of the outbreaks.

The recommendations that can be given based on this work include closely supervised procedures for appropriate use of PPE, as having problems with the PPE was identified as a risk factor. We also observed that several students struggled to remove the PPE, at least partly because of the use of duct tape, a predicament that could increase the dispersion of the oocysts and the risk of infection. The possibility of using another type of gown could be considered. Additionally, strict adherence to the use of soap for hand washing, which appeared as a protective factor, should be emphasized, together with highlighting that alcohol gels do not inactivate *Cryptosporidium* oocysts (19). Several students stated that they did not use soap after each of the steps—PPE removal, washing boots, and changing to private clothing—although all stated using soap at least once. This was unexpected because handwashing with soap after clinical work is a standard procedure taught during clinical training at the veterinary hospitals.

Recommendations to improve the facilities were also given, but having designated areas for clean PPE storage, donning, and handwashing proved to be insufficient alone to prevent new cases of cryptosporidiosis. In future investigations, swab samples from the room surfaces (e.g., the fetotomy boxes or the sinks) and the PPE (e.g., masks and boots) could be useful to evaluate the timing and extent of oocyst contamination, which could occur at any point from the beginning of the exercise until cleaning of the room with pressurized water.

Outbreaks of cryptosporidiosis have become a matter of concern in the academic training of veterinary students. In our study, an increased awareness among the students might have been in place, compared with other settings where fewer protective measures are used and infections among the students have happened as well (24). Several students emphasized their interest in the exercise, but more than two thirds of the students declared nervousness about becoming sick in relation to the fetotomy exercise, and some students were observed wearing two face masks or four pairs of gloves on top of each other. This is in line with previous conceptions of enteric disease being a “rite of passage” in the veterinary curriculum, while nowadays the level of concern is higher (12).

Several measures can be implemented to decrease the risk of cryptosporidiosis among veterinary students (12), including reducing the involvement of veterinary students with animals, which has, however, been suggested with the remark “must be balanced with the value of clinical experience in producing practice-ready new graduates” (12). It is important to prevent outbreaks of zoonotic infectious diseases while ensuring proper training of the veterinary students. Due to the exposure of veterinary students to zoonoses (24), surveillance and reporting systems at the universities would be beneficial to reduce the risk of transmission of zoonotic pathogens to this risk group and potential secondary cases (25).

## MATERIALS AND METHODS

### Setting.

The University of Copenhagen is the only university in Denmark providing the education of veterinarians. As part of the education, 4th-year veterinary students, distributed in groups of approximately 10 students, attend a fetotomy exercise over two subsequent days throughout the academic year. The dates shown in Fig. 1 and 3 refer to the first day of each exercise.

In the beginning of the exercise, the students are involved in the handling and euthanizing (by intravenous application of an overdose of barbiturate) of the calves to be used. Next, the supervisor removes the gastrointestinal tract of each calf to reduce exposure to intestinal content during the exercise. After demonstration of the standard fetotomy procedures (26) (Text S1), the students are divided into subgroups of 3 or 4 students, and each subgroup performs hands-on exercises on a euthanized calf. In the present study, all students were considered at equal risk of infection, as all the groups were working in the same setting, a single class I laboratory-equivalent exercise room.

The calves were young males bought live from a convenience selection of farms in the morning of the first day of the exercise. The farms were instructed to provide calves as young as possible so as to have them resemble the natural size of a calf at the time of birth.

### Timeline of investigation and study design.

Our investigation was initiated following an outbreak in September 2018. The timeline of the investigation was separated into a retrospective period (I) and a prospective study (periods II and III) (Fig. 1, Table S1). For period I (January to August 2018), information on symptoms compatible with cryptosporidiosis reported by students was retrieved retrospectively from records of the University of Copenhagen. The prospective cohort study comprised period II (September 2018 to January 2019) and period III (February to June 2019). Ten days after each exercise, a link to an online questionnaire (Text S2) was sent by email to all students in the group, and students who had symptoms were encouraged to submit a stool sample for testing. The key difference between period II and period III was that during period II, the training was compulsory, while in period III, it was voluntary (Table S1). The prospective study included two site visits to observe the exercises and the facilities in order to propose and observe interventions that could serve to minimize the risk of infection in future exercises.

### Microbiological investigations.

Fecal samples from all calves used during periods I to III were collected from the rectum or caudal colon during the removal of the gastrointestinal tract and stored at −20°C until analysis. Stool samples from students were processed when received in the laboratory. Genomic DNA from all samples was extracted with a NucliSENS easyMag platform (bioMérieux, Durham, NC, USA) and analyzed with an in-house, duplex real-time PCR for detection of *Cryptosporidium* spp. and Giardia duodenalis using a cycle threshold (*C_T_*) value of <40 as the cutoff value for positivity. The stool samples from the outbreak that occurred in January 2019 were, moreover, tested using real-time PCR for enteropathogenic Clostridium perfringens, Bacillus cereus, norovirus, rotavirus, sapovirus, astrovirus, and adenovirus (details not shown).

Subtyping with conventional nested PCR targeting the *gp60* locus (27) was carried out for samples positive for *Cryptosporidium*. Multiple alignment of the *gp60* sequences against representative sequences of different *gp60* subtypes (7) was carried out using MEGA X (28).

The *Cryptosporidium* load in the samples from the calves was used as a proxy for the level of infection and shedding. Based on the *C_T_* values, three categories were established; the quartile of positive calves with the largest amount of *Cryptosporidium* DNA detected (i.e., lowest *C_T_* values) was considered “highly positive” (+++); the quartile with the smallest amount of DNA (i.e., highest *C_T_* values) was considered “weakly positive” (+), and the intermediate quartiles were considered “positive” (++).

### Case definition.

Gastrointestinal symptoms (diarrhea, vomiting, abdominal cramps, and/or nausea) with or without fever occurring within 2 to 12 days after the exercise were considered compatible with cryptosporidiosis. A laboratory-confirmed case was defined as a student who (i) participated in a fetotomy exercise between September 2018 and June 2019, (ii) reported compatible symptoms through the questionnaire, and (iii) tested positive for *Cryptosporidium* spp. A probable case was a student fulfilling points i and ii but without laboratory confirmation of the causative agent being *Cryptosporidium* spp. A suspected case was a student fulfilling point i who had informed the university staff of compatible symptoms. Any cases from outbreaks that occurred in period I (January to August 2018), identified retrospectively, were considered suspected cases.

### Statistical analysis.

Risk ratios (RR), 95% confidence intervals (CI), and probability (*P*) values were calculated using Fisher’s exact test. For the statistical analysis, calves were categorized into the following age groups: <7, 7 to 13, 14 to 20, and >20 days old; these categories were dichotomized to <7 and ≥7 days old for the statistical analysis. Stata 14 (Stata Statistical Software release 14 [2015], StataCorp LP, College Station, TX, USA) and Microsoft Excel 2013 were used for the analyses.

### Data sources, data availability, and ethical considerations.

Dates of the fetotomy exercises and student contact information were obtained from the University of Copenhagen. Information on which calves had been used by which subgroup of veterinary students was available for each of the exercises. Information on the age of the calves was obtained from unique ear tag numbers through the Danish Livestock Register (https://chr.fvst.dk/).

The calves were handled according to high ethical standards and national legislation. Approval was provided by the Local Ethical and Administrative Committee of the Department of Veterinary Clinical Sciences, University of Copenhagen (2020-011). The calves were not regarded as experimental animals according to the Danish animal welfare legislation.

The students were contacted by email by the outbreak investigation team. The students were informed about the purpose of the investigation and that the collected data were handled confidentially and presented in an anonymized and aggregated format.

**Data availability.** Representative *gp60* sequences were submitted to GenBank under accession numbers MT472732 (sequence from a case sample) and MT472731 (sequence from a calf sample).

## Supplementary Material

Supplemental file 1
