# Supplementary material for: Veterinary Students Have a Higher Risk of Contracting Cryptosporidiosis when Calves with High Fecal Cryptosporidium Loads Are Used for Fetotomy Exercises
Source: Appl Environ Microbiol. 2020 Sep 17;86(19):e01250-20. doi: 10.1128/AEM.01250-20 (PMC7499042; doi:10.1128/AEM.01250-20)
Supplement: Supplemental file 1 [file AEM.01250-20-s0001.pdf]

**SUPPLEMENTAL INFORMATION**

# Veterinary Students Have A Higher Risk Of Contracting Cryptosporidiosis When Calves With High Fecal Cryptosporidium Loads Are Used For Fetotomy Exercises

Daniel Thomas-Lopez[a,b,#], Luise Müller[c], Lasse S. Vestergaard[b,c], Mette Christoffersen[d], Anne-Marie Andersen[c], Pikka Jokelainen[b], Jørgen Steen Agerholm[d], Christen Rune Stensvold[b]

[a] European Public Health Microbiology Training Programme (EUPHEM), European Centre for Disease Prevention and Control (ECDC), Stockholm, Sweden

[b] Laboratory of Parasitology, Department of Bacteria, Parasites & Fungi, Infectious Disease Preparedness, Statens Serum Institut, Copenhagen, Denmark

[c] Department of Infectious Disease Epidemiology and Prevention, Infectious Disease Preparedness, Statens Serum Institut, Copenhagen, Denmark

[d] Department of Veterinary Clinical Sciences, University of Copenhagen, Taastrup, Denmark

[#] Address correspondence to Daniel Thomas-Lopez, DATL@ssi.dk

Contents:

Figure S1

Table S1

Table S2

Text S1

Text S2

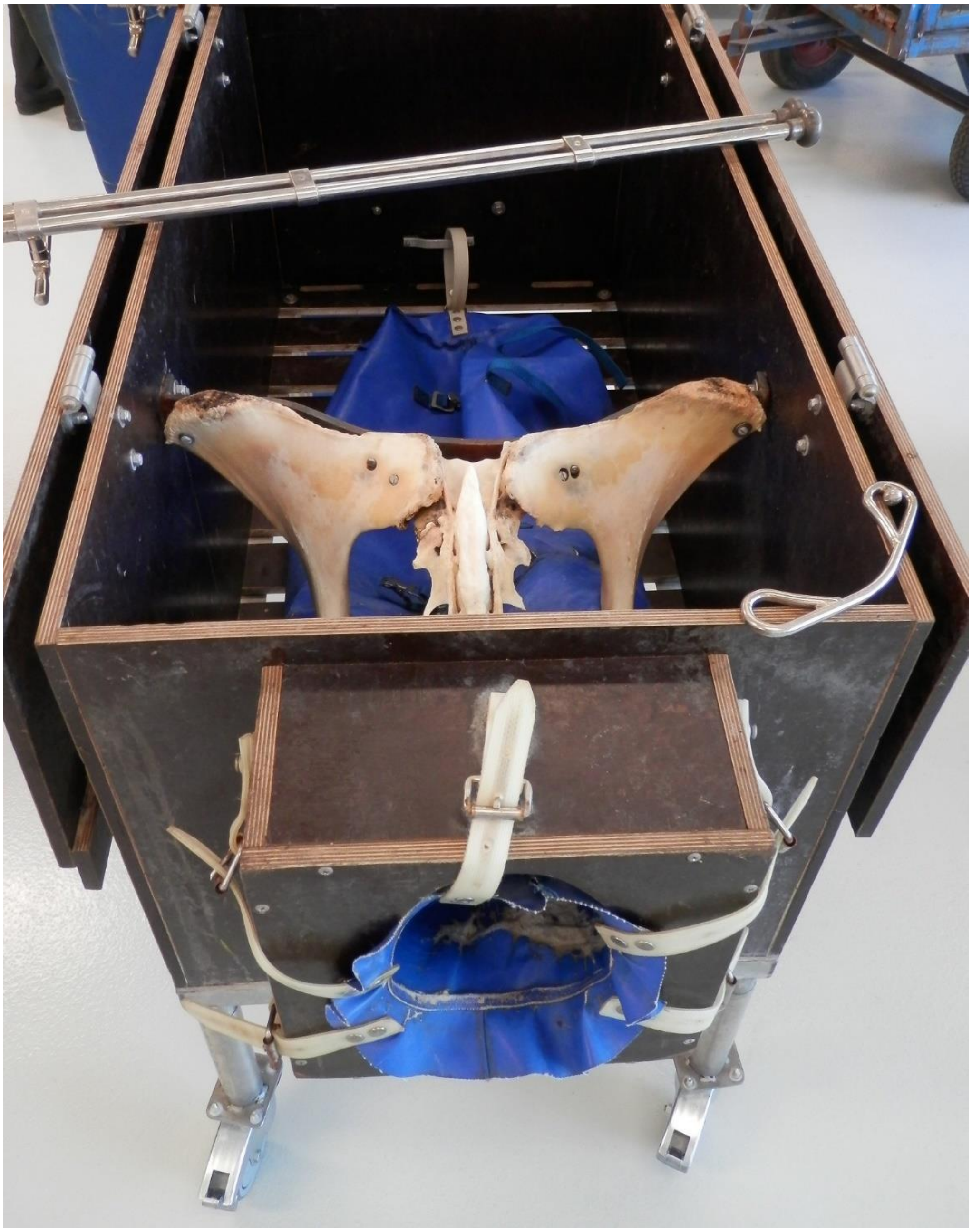

**Figure S1.** Fetotomy box containing an artificial uterus (blue bag) inside of which the euthanized calf is placed, and an opening through which the euthanized calf is dissected and extracted. Fetotomy exercises, University of Copenhagen, Denmark, 2018-2019.

**Table S1. Descriptive characteristics of the three periods of time in the study.**

| | **Period I** | **Period II** | **Period III** | **Total** |
|---|---|---|---|---|
| **Investigation** | Retrospective | Prospective | Prospective | |
| **Information on fetotomy exercises and students** | | | | |
| **Number of fetotomy exercises** | 9 | 8 | 8 | 25 |
| **Attendance to a fetotomy exercise** | Mandatory | Mandatory | Voluntary | |
| **Number of students registered for the fetotomy exercises** | Not retrieved | 88 | 68 | 156 |
| **Number of students attending the fetotomy exercises** | Not retrieved | 87 | 31 | 119 |
| **Cohort study (questionnaire)** | No | Yes | Yes | |
| **Number of laboratory-confirmed cases** | NA | 5 | 0 | 5 |
| **Number of probable cases** | NA | 6 | 0 | 6 |
| **Number of suspected cases** | 5 | 1 | 0 | 6 |
| **Information on calves** | | | | |
| **Fecal samples collected from calves** | Yes | Yes | Yes | |
| **Number of calves used for fetotomy exercises** | 45 | 37[a] | 11 | 93[a] |
| **Number (%) of calves positive for *Cryptosporidium* by real-time PCR** | 25 (56%) | 22 (59%) | 6 (55%) | 53 (57%) |

[a]Microbiological information on one of the samples was not available and is not included here

NA, Not applicable

**Table S2. Univariable analysis for intervention and risk factors included in the questionnaire for the students participating in the fetotomy exercises at the University of Copenhagen, Denmark, from September 2018 to June 2019 (N = 97)[a,b]**

| Factor | Factor present | No. of cases | Total[c] | AR (%) | RR | 95% CI | *P* value |
|---|---|---|---|---|---|---|---|
| **Intervention: designated areas for PPE storage, donning and handwashing** | Yes | 7 | 75 | 9.33 | 0.51 | 0.17-1.59 | 0.264 |
| | No | 4 | 22 | 18.18 | | | |
| **Q1. Interaction with calves:** | **Factor present** | **No. of cases** | **Total[c]** | **AR (%)** | **RR** | **95% CI** | ***P* value** |
| **Had PPE visibly contaminated with feces** | Yes | 8 | 29 | 27.59 | **11.31** | **1.49-85.58** | **0.003** |
| | No | 1 | 41 | 2.44 | | | |
| **Dissected a calf with visible diarrhea** | Yes | 9 | 48 | 18.75 | **NA** | **NA** | **0.023** |
| | No | 0 | 25 | 0 | | | |
| **Carried a calf** | Yes | 7 | 51 | 13.73 | 1.58 | 0.49-5.05 | 0.531 |
| | No | 4 | 46 | 8.70 | | | |
| **Hold a calf** | Yes | 9 | 71 | 12.68 | 1.58 | 0.37-6.84 | 0.723 |
| | No | 2 | 25 | 8 | | | |
| **Euthanized a calf** | Yes | 6 | 50 | 12 | 1.13 | 0.37-3.45 | 1 |
| | No | 5 | 47 | 10.64 | | | |
| **Touched calves while not wearing gloves** | Yes | 0 | 4 | 0 | 0 | NA | 1 |
| | No | 11 | 90 | 12.22 | | | |
| **Q2. Personal Protective equipment (PPE):** | **Factor present** | **No. of cases** | **Total[c]** | **AR (%)** | **RR** | **95% CI** | ***P* value** |
| **Experienced problems with the PPE** | Yes | 9 | 48 | 18.75 | **4.59** | **1.05-20.17** | **0.028** |
| | No | 2 | 49 | 4.08 | | | |
| **Used duct tape at the start of the exercise** | Yes | 11 | 80 | 13.75 | NA | NA | 0.203 |
| | No | 0 | 17 | 0 | | | |
| **Used own glasses** | Yes | 0 | 10 | 0 | 0 | NA | 0.343 |
| | No | 9 | 62 | 14.52 | | | |
| **Used mask** | Yes | 10 | 87 | 11.49 | 0.69 | 0.11-4.53 | 0.541 |
| | No | 1 | 6 | 16.67 | | | |
| **Used 3M mask** | Yes | 1 | 8 | 12.50 | 1.48 | 0.20-11.08 | 0.549 |
| | No | 5 | 59 | 8.47 | | | |
| **Used duct tape during the exercise** | Yes | 1 | 18 | 5.56 | 0.44 | 0.06-3.21 | 0.683 |
| | No | 10 | 79 | 12.66 | | | |
| **Did not use duct tape** | Yes | 0 | 4 | 0 | 0 | NA | 1 |
| | No | 11 | 93 | 11.83 | | | |
| **Had boots with laces** | Yes | 0 | 4 | 0 | 0 | NA | 1 |
| | No | 11 | 93 | 11.83 | | | |
| **Used long gloves** | Yes | 11 | 95 | 11.58 | NA | NA | 1 |

| | | | | | | | |
|---|---|---|---|---|---|---|---|
| | No | 0 | 1 | 0 | | | |
| **Used cap** | Yes<br>No | 11<br>0 | 94<br>1 | 11.70<br>0 | NA | NA | 1 |
| **Used short gloves** | Yes<br>No | 8<br>0 | 82<br>0 | 9.76<br>0 | NA | NA | NA |
| **Used boots** | Yes<br>No | 11<br>0 | 97<br>0 | 11.34<br>0 | NA | NA | NA |
| **Used disposable gown** | Yes<br>No | 11<br>0 | 97<br>0 | 11.34<br>0 | NA | NA | NA |
| **Q3. Behavior during the exercise:** | **Factor present** | **No. of cases** | **Total[c]** | **AR (%)** | **RR** | **95% CI** | ***P* value** |
| **Ate after the exercise** | Yes<br>No | 0<br>11 | 16<br>81 | 0<br>13.58 | 0 | NA | 0.202 |
| **Used the mobile phone after the exercise** | Yes<br>No | 2<br>6 | 42<br>49 | 4.76<br>12.24 | 0.39 | 0.08-1.83 | 0.279 |
| **Wore earrings** | Yes<br>No | 2<br>9 | 26<br>70 | 7.69<br>12.86 | 0.6 | 0.14-2.59 | 0.722 |
| **Left the room during the exercise** | Yes<br>No | 1<br>10 | 13<br>83 | 7.69<br>12.05 | 0.64 | 0.09-4.58 | 1 |
| **Used mobile phone during the exercise** | Yes<br>No | 0<br>11 | 2<br>95 | 0<br>11.58 | 0 | NA | 1 |
| **Smoked during the exercise[d]** | Yes<br>No | 0<br>11 | 2<br>95 | 0<br>11.58 | 0 | NA | 1 |
| **Ate during the exercise[d]** | Yes<br>No | 0<br>11 | 3<br>94 | 0<br>11.70 | 0 | NA | 1 |
| **Wore ring** | Yes<br>No | 0<br>11 | 0<br>96 | NA<br>11.46 | NA | NA | NA |
| **Wore bracelet** | Yes<br>No | 0<br>11 | 0<br>96 | NA<br>11.46 | NA | NA | NA |
| **Q4. Hand hygiene after doffing disposable gown and long gloves[e]:** | **Factor present** | **No. of cases** | **Total[c]** | **AR (%)** | **RR** | **95% CI** | ***P* value** |
| **Used soap and alcohol gel** | Yes<br>No | 3<br>7 | 49<br>24 | 6.12<br>29.17 | **0.21** | **0.06-0.74** | **0.012** |
| **Used only soap** | Yes<br>No | 0<br>7 | 15<br>24 | 0<br>29.17 | **0** | **NA** | **0.031** |
| **Used only alcohol gel** | Yes<br>No | 0<br>7 | 2<br>24 | 0<br>29.17 | 0 | NA | 1 |
| **Q5. Hand hygiene after washing boots and doffing short gloves[e]:** | **Factor present** | **No. of cases** | **Total[c]** | **AR (%)** | **RR** | **95% CI** | ***P* value** |
| **Used soap and alcohol gel** | Yes<br>No | 8<br>1 | 72<br>5 | 11.11<br>20 | 0.56 | 0.09-3.61 | 0.472 |
| **Used only soap** | Yes<br>No | 1<br>1 | 7<br>5 | 14.29<br>20 | 0.71 | 0.06-8.90 | 1 |

| | | | | | | | |
|---|---|---|---|---|---|---|---|
| **Used only alcohol gel** | Yes<br>No | 1<br>1 | 4<br>5 | 25<br>20 | 1.25 | 0.11-14.34 | 1 |
| **Q6. Hand hygiene after doffing cap and mask[e]:** | **Factor present** | **No. of cases** | **Total[c]** | **AR (%)** | **RR** | **95% CI** | ***P* value** |
| **Used soap and alcohol gel** | Yes<br>No | 7<br>1 | 60<br>10 | 11.67<br>10 | 1.17 | 0.16-8.50 | 1 |
| **Used only soap** | Yes<br>No | 0<br>1 | 2<br>10 | 0<br>10 | 0 | NA | 1 |
| **Used only alcohol gel** | Yes<br>No | 3<br>1 | 12<br>10 | 25<br>10 | 2.5 | 0.31-20.45 | 0.594 |
| **Q7. Hand hygiene after changing clothes:** | **Factor present** | **No. of cases** | **Total[c]** | **AR (%)** | **RR** | **95% CI** | ***P* value** |
| **Used soap and alcohol gel** | Yes<br>No | 11<br>0 | 80<br>4 | 13.75<br>0 | NA | NA | 1 |
| **Used only soap** | Yes<br>No | 0<br>0 | 3<br>4 | 0<br>0 | NA | NA | NA |
| **Used only alcohol gel** | Yes<br>No | 0<br>0 | 6<br>4 | 0<br>0 | NA | NA | NA |
| **Q8. Hygiene after the exercise:** | **Factor present** | **No. of cases** | **Total[c]** | **AR (%)** | **RR** | **95% CI** | ***P* value** |
| **Reused the clothes without washing** | Yes<br>No | 4<br>7 | 17<br>80 | 23.53<br>8.75 | 2.69 | 0.88-8.17 | 0.098 |
| **Put the clothes in the locker** | Yes<br>No | 0<br>11 | 16<br>80 | 0<br>13.75 | 0 | NA | 0.202 |
| **Took a shower immediately afterwards** | Yes<br>No | 2<br>9 | 34<br>63 | 5.88<br>14.29 | 0.41 | 0.09-1.80 | 0.319 |
| **Changed clothes right after the exercise** | Yes<br>No | 11<br>0 | 86<br>11 | 12.79<br>0 | NA | NA | 0.354 |
| **Kept the same clothes on for the rest of the day** | Yes<br>No | 0<br>11 | 2<br>94 | 0<br>11.70 | 0 | NA | 1 |
| **Put the clothes in the handbag** | Yes<br>No | 0<br>11 | 2<br>93 | 0<br>11.83 | 0 | NA | 1 |
| **Put the clothes in a plastic bag** | Yes<br>No | 10<br>1 | 83<br>13 | 12.05<br>7.69 | 1.57 | 0.22-11.24 | 1 |

[a]AR, attack rate; RR, risk ratio; CI, confidence interval; PPE, personal protective equipment; Q, question number; NA, not applicable.

[b]Risk factors for each question are ranked by increasing P value.

[c]Those who replied “I don’t know / I don’t remember” were omitted from the analysis for the specific determinant

[d]Did not take place in the exercise room

[e]Questions potentially ambiguous as specific order of doffing was not stated

## Text S1. The fetotomy procedure

Each group of students performed two standard fetotomies according to Vermunt (1). On day 1, fetotomy on a calf in anterior longitudinal presentation was done using a Thygesen's fetotome and multifilament fetotomy wire (Bovi-vet, Kruuse, Langeskov, Denmark). The final cut line of this procedure goes through the pelvis and the remnants of the rectum. On day 2, fetotomy on a calf in posterior longitudinal presentation was done. The first cut line of this procedure goes through the pelvis and the remnants of the rectum.

## Text S2. Online questionnaire

The questionnaire, designed in Enalyzer (https://www.enalyzer.com/), included multiple choice and open questions on clinical aspects (e.g. symptoms, duration, and medical care) and questions to identify possible risk factors, such as contact with the calves (e.g. carrying or handling a calf with visible diarrhea), personal protective equipment (PPE) used, hygiene measures (hand washing, showering after the exercise), management of the clothes (storage, washing temperature), eating and/or drinking during and after the exercise, smoking or use of mobile phone. The students were also asked about awareness and information received from the teacher prior to the exercise on the risk of cryptosporidiosis, as well as about being anxious about acquiring cryptosporidiosis, and whether the student had other recent contact with calves.

For Period III, questions about risk factors and behaviors were received only from those who voluntarily participated in the hands on exercise, while general demographics, open text answers and comments were provided both by participants and by non-participants who received the questionnaire.

After initial sending of the questionnaire, if needed up to two reminders were sent, 10 and 20 days after the first email.

## References


1. Vermunt J. 2009. Fetotomy, p 326–343. *In* Noakes D, Parkinson T, England G (ed), Veterinary Reproduction and Obstetrics, 9th ed. Saunders Elsevier, Oxford, Saunders Co. Ltd, London, United Kingdom.
